# ‘Please, see me’; Informal and professional support of students with relatives with addiction problems: a three-year longitudinal qualitative study

**DOI:** 10.1186/s12889-024-20531-8

**Published:** 2024-11-08

**Authors:** Dorine M. van Namen, Sander R. Hilberink, Hein de Vries, Gera E. Nagelhout, AnneLoes van Staa

**Affiliations:** 1 Research Center Innovations in Care, Rotterdam University of Applied Sciences, Rotterdam, The Netherlands; 2 Department of Health Promotion, CAPHRI Care and Public Health Research Institute, Maastricht University, Maastricht, The Netherlands; 3 Centre of Expertise Perspective in Health, Avans University of Applied Sciences, Breda, the Netherlands

**Keywords:** Affected family members, Adult children of alcoholics, Addiction, Mental Health, Support, Students, Longitudinal qualitative research

## Abstract

**Background and aim:**

Addiction problems also affect the lives of family members. This study aims to examine: (1) young adult family members’ experiences with informal and professional support in coping with the impact of relatives’ addiction problems and (2) how these experiences evolve over time.

**Method:**

A three-year longitudinal qualitative study. Four rounds of in-depth, semi-structured individual interviews were conducted. Thirty students aged 18–30 years, participated in the study at baseline. 93% participated in at least two interviews, and 80% participated three or four times. The Stress-Strain-Information-Coping-Support model was used, and Directed Content Analysis was applied.

**Findings:**

Five major themes were extracted from the data: (1) Informal support; (2) Educational support; (3) Healthcare support; (4) Resilience factors; and (5) Developments over time. Informal and educational support were more often described as effective than healthcare support, although the number of participants who sought healthcare support increased over time. Effective elements of support included being able to discuss their experiences with people listening without judgment or unsolicited advice and having long-term relationships of trust with people from the social environment and professionals. Participants were mainly treated with Cognitive Behavioral Therapy (CBT) and Eye Movement Desensitization and Reprocessing (EMDR). Learning how to distinguish between accurate and inaccurate thoughts, especially about themselves, was considered effective. Body-oriented therapy was remarkably absent. Finding effective healthcare support was often a long and winding road through various therapies and therapists. Participants were not attracted to peer group interventions but needed advice on how to deal with their relatives. They also needed recognition by their relatives for harm done. This recognition was seldom given.

**Conclusions:**

It is recommended to train educational and healthcare professionals to recognize the support needs of young people with relatives with addiction problems, to help them cope, or to refer them adequately. We also suggest broadening the scope of professional support offered to AFMs, including body-oriented and cultural interventions.

**Supplementary Information:**

The online version contains supplementary material available at 10.1186/s12889-024-20531-8.

## Introduction

Family members of people with addiction problems (referred to as Affected Family Members (AFMs)) are a high-risk group for the development of health, social, and financial problems [1–3]. The focus of research on AFMs’ experiences is primarily on partners, parents, and young children [2]. Research into harm experienced by young adult family members is limited and focuses mainly on the young adults’ own alcohol and drug problems [4]. 75% of mental disorders emerge before the age of 24 years [5]. Worldwide, violence and self-inflicted injuries are – after traffic accidents and HIV/Aids – the third leading cause of death in young people aged 20–24 years [5, 6]. In the Netherlands suicide is the leading cause of death for young adults between 20 and 24 years old [7]. Young adult AFMs are a group at risk for these severe problems [8].

Furthermore, the number of young adult AFMs is high. In our study, we found that 15.6% of the students of Rotterdam University of Applied Sciences (RUAS) had relatives with addiction problems [3]. These students experienced high levels of aggression: physical violence (such as kicking or throwing down the stairs) or emotional violence (such as humiliating, insulting, and belittling), suffered from neglect and negative behavior from their relatives (such as lying and manipulating), experienced addiction-related death, illnesses, and accidents, and had more family caregiving responsibilities than students without relatives with addiction problems. As a result, these young adult AFMs suffered from a wide range of physical complaints and pain, sleeping problems, and mental health problems such as depression, anxiety, low self-esteem, and self-harm (including suicide ideation and suicide attempts) [8].

Protective factors can reduce the likelihood of poor outcomes for AFMs and increase their resilience. Although there is no single definition of resilience, most definitions involve the process of adapting to and bouncing back from adversity [9]. There are several protective factors. First, individual factors such as personal qualities, self-acceptance, social skills, having a hobby or talent, making plans and intelligence, can protect an individual from the negative consequences of life with relatives with addiction problems [10–12]. Second, family factors, such as having a close relationship with at least one stable family member, or sufficient finances can contribute to resilience. Third, AFMs may be protected by community factors, such as support from teachers, and neighbors, or active participation in religious activities and rituals [10–13]. Resilience is complex. Protective factors vary over time and are influenced by the severity of risk factors, age, development, gender, and culture. There are no one-to-one relationships between risk and resilience factors. They manifest themselves in a complex balance [12].

**Fig. 1:**
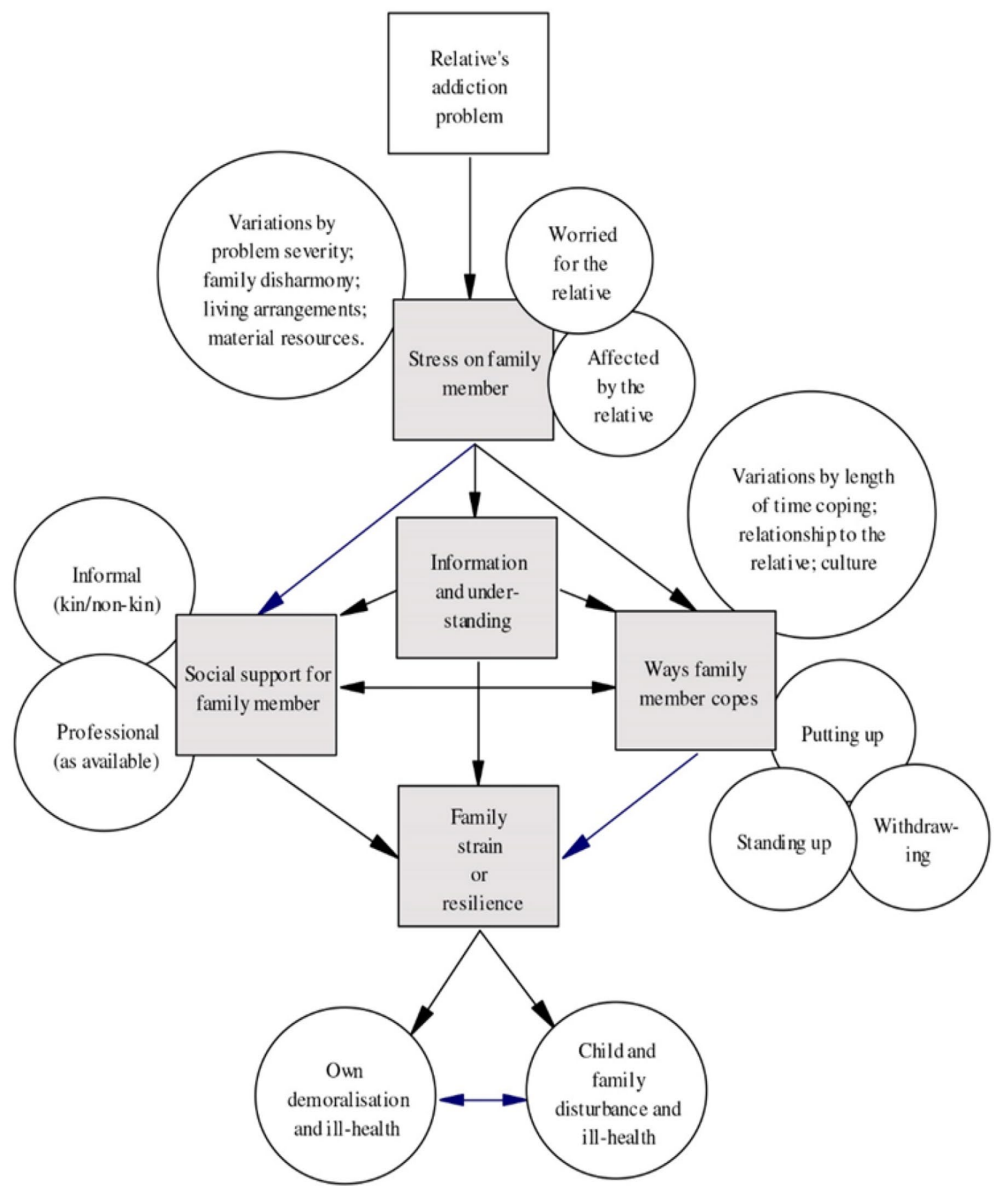
Stress-Strain-Information-Coping-Support-model

The Stress-Strain-Information-Coping-Support-model (SSICS) (Fig. 1) describes and explains the experiences of AFMs [2].

This model assumes that having a relative with addiction problems is highly stressful and causes strain on the family member [2]. The SSICS-model posits that AFM’s stress and strain are mediated by the positive or negative influence of the coping strategies they use and the extent and quality of the support they receive. Support includes informal and professional support [2].

Professional support mainly focuses on those with addiction problems, not their family members [2, 14]. Interventions primarily oriented toward the needs of AFMs are fragmented and limited in many countries [2, 15]. There is hardly any information regarding the number of AFMs reached, the treatment procedures used, and the scope of possible support. Exceptions tend to be Denmark and Sweden with a tax-financed care system in which the care of AFMs is an integral part of addiction support [16]. In the Netherlands, there is no general national policy for support to AFMs. The need for this type of professional support is recognized in the National Prevention Agreement of 2018 (an agreement to make the Dutch healthier by reducing smoking, problematic alcohol use, and obesity), which should have been implemented in 2020 [17]. However, this target has not been met. Yet, there are two effective interventions for AFMs in the Netherlands. The 5-Step Method – offered by one organization – is a simple, short, semi-structured psychosocial intervention for AFMs in their own right. The 5-Step Method can be implemented by trained practitioners (laypersons or people with relevant professional qualifications) and is given to individuals, couples, or groups [18]. Community Reinforcement and Family Training (CRAFT) is a structured intervention for AFMs to promote their well-being, improve their coping behaviors, and encourage their relatives to enter treatment [19]. This intervention is offered in a few places in the Netherlands. Also, most addiction care institutions have family programs, which offer information sessions for family members about addiction and sometimes peer-to-peer contact during treatment of the person with addiction. The primary aim of these programs is for AFMs to support their relatives in their recovery process [20–22]. Unfortunately, only 10% of all persons with addiction problems enter addiction treatment [23] indicating that most family members remain out of sight of addiction services.

Every Dutch municipality has a legal obligation to offer a support program for Children Of Parents with Mental Illness and/or addiction (COPMI) up to 25 years, the so-called COPMI groups [24]. Outreach to these groups is low and there is a lack of a structured and integrated approach to identifying and supporting COPMI [25]. Organizations providing mental health or addiction services to parents can play an important role in identifying children at risk and referring them to COPMI groups. However, this does not happen often [26], whereas early and preventive interventions could reduce the risk of developing problems in children and adolescents by 40% [27, 28].

Little research has been done on the support experiences of young adult AFMs [29–32]. Therefore, this study aims to examine (1) young adult AFMs’ experiences with informal and professional support in coping with the impact of relatives’ addiction problems and (2) how these experiences evolve over time.

## Method

### Design

This study is part of a three-year longitudinal qualitative study consisting of in-depth, individual semi-structured interviews with students in higher education in the Netherlands who have relatives with addiction problems [33]. Longitudinal research among AFMs is rare and concerns mainly quantitative studies of the development of substance use problems among AFMs themselves [34]. Longitudinal Qualitative Research (LQR) is suitable when researching transitions in life course issues, transitions to adulthood, family lives, and the impact of key life events [35]. We interviewed the students once a year from the end of 2019/beginning of 2020 to the end of 2021-beginning 2022. Additionally, we interviewed some of them in the spring of 2020, when the COVID-19 pandemic just broke out.

### Participant recruitment

Students at RUAS who had participated in an online survey on their own substance use [3] were invited to the present study. The survey also included questions about their relatives’ substance use and was used as a screener for our qualitative study [33]. Eligible students for the qualitative interviews were those who (1) responded positively to the question ‘Is there anyone in your family with behavioral and/or health problems caused by alcohol/drugs/medication, such as painkillers, sleeping medications, or tranquilizers?’ (*n* = 881), and (2) provided contact details (*n* = 105 of 881) [33]. A study coach from the Hague University of Applied Sciences (HUAS) posted a reference to our study on the university’s intranet, which led to four students signing up for the qualitative interviews. Students older than 30 years were excluded. We then applied purposive sampling [36], aiming for an equal distribution of genders, and to compose a diverse group based on the study program, study year, type of substance use of the relative, and relationship to relatives [33].

### Participants

Thirty students were included for interviews in the first year: Twenty-eight from RUAS and two from HUAS. In total, 28 students (93%) participated in at least two interviews, and 24 (80%) participated in three or four interviews. Reasons given for withdrawing from the study were: being too busy after graduation, not wanting to stir up difficult family relationships anymore, having been admitted to a clinic for serious mental health problems, or the death of a relative with addiction problems [33]. Of the original 30 participants (mean age 22.9, SD 2.7), 19 were women and 11 were men (Table 1) [8].

**Table 1:** Characteristics of participants at baseline

	Baseline *N* = 30
**Age in years**	
17–20 21–24 25–30 *Mean (SD)*	4 20 6 22.9 (2.7)
**Study program**	
Economics Social studies Healthcare Arts Media and ict Teacher training Engineering	5 7 5 2 5 3 3
**Study delay (yes)**	11
**Mental health diagnosis (yes)**	18
**Lives in the same house as relative with addiction (yes)**	9
**Relative in treatment (in addiction care)**	2
**Relative died due to drinking and/or drugs**	3
Number of relative(s) with addiction in nuclear family*	
1 relative 2 relatives 3 relatives 4 relatives	10 14 4 2
Number of relative(s) with addiction in extended family*	
1 relative 2 relatives 3 relatives 4 relatives ≥ 5 relatives	8 5 3 3 2
**Total number of relatives with addiction in the family**	
1 relative 2 relatives 3 relatives 4 relatives ≥ 5 relatives	3 11 2 4 10
Relationship with relative with addiction in nuclear family**	
Father Mother Stepmother Stepfather (Step)siblings (Ex-)partner	14 13 1 6 12 5
**Type of relative’s addiction in nuclear family**	
Alcohol only Drugs only Poly use (combinations of alcohol, drugs, and/or tranquilizers; gambling and/or sex addiction)	11 1 18
**Ethnic background**	
Dutch From Suriname and Netherlands Antilles From another Western country	26 2 2

* All sorts of combinations occurred: having one relative with addiction in the nuclear family and two in the extended family or for example two in the nuclear family and none in the extended family

** Participants can have more than one relative with an addiction [8]

### Interview process

The interview topic list (Appendix) was derived from the SSICS-model [2]. It contained open-ended questions based on the main categories of the SSICS-model (Stress, Strain on participants’ health and social life, Information, Coping, and Support), supplemented by questions about study experiences [33]. In this paper, we focus on the component ‘support’ of the SSICS-model: Did participants seek support? Did they receive support? From whom? When was support effective? When was it not effective? What support needs did participants have? The same questions were asked during the subsequent interview rounds, to detect change. All interviews were conducted by the same researcher (DvN).

The interviews in the first round were conducted in person and lasted on average 78 min (range 53–116 min). The interviews were conducted at the university premises or in a public location, according to the preference of the participant. The interviews in the additional interview round during the first COVID-19 lockdown were conducted online and lasted on average 23 min (range 15–33 min). For online interviews, the videoconferencing platform Microsoft Teams was used. The interviews in the second and third years were conducted either online or live (depending on the interviewee’s preference) and lasted on average 78 min (range 38–133 min). Face-to-face interviews were conducted at interviewees’ homes, at the university premises, or in a public location, depending on the interviewee’s preference. At the end of each interview and the beginning of follow-up interviews, the participant received a verbal summary of the key findings from their (previous) interview for participant checking [33]. Data collected at all four timepoints were used for this paper.

In between interviews, the interviewer maintained personal contact with the participants through email and messaging app WhatsApp, on average twice a year. Interviews were recorded digitally, transcribed verbatim, and pseudonymized for analyses. Transcriptions were done by a student not affiliated with the universities involved, to prevent recognition of a fellow student. This student signed a declaration of confidentiality. Participants were informed (verbally and in writing) about the aim of the research and the procedures (voluntary participation, anonymity), including the fact that they could end their cooperation at any time. Participants were not compensated but received a small gift at the end of the study. All participants signed an informed consent statement [33].

### Data analysis

We applied synchronic analyses (cross-sectional after each interview round) with the Directed Content Analysis procedure and diachronic analysis (change over time) after four interview rounds. Directed Content Analysis is used for qualitative analysis to validate, refine, and/or extend a theory in a new context [37, 38]. Seven steps were followed [33, 37]:

1. Two researchers were immersed in the data (DvN and a research assistant).
2. Before coding, a formative matrix of the main categories (Stress-Strain-Information-Coping-Support) and related subcategories (for example Informal support: family; friends; partner; work; other) was deductively derived from the existing theory and previous research.
3. The main categories were defined and supplemented by examples.
4. Anchor samples for each main category were added.
5. The first-round interviews were then coded by the two aforementioned researchers independently. They read and reviewed the transcripts several times, and discussed the coding, the categorization matrix, and any disagreements until an agreement was reached.
6. New codes, retrieved inductively from the data, were added (for example, educational support: secondary school; university; internships).
7. Codes were organized in an iterative process using summary tables. The analysis outcomes were discussed by the entire research team, after which some refinements and additions were made [8].

This led to a final codebook with 58 codes. Subsequent follow-up interviews were coded by the first author using the code book of the first round, which then was supplemented with new codes, especially about the impact of the COVID-19 pandemic. The final codebook comprised 62 codes. The analysis and coding processes of all follow-up interviews were critically reviewed by a second researcher (AvS) and followed by a discussion to resolve disagreements about coding. These analysis outcomes were then discussed by the entire research team, after which some refinements and additions were made. Data analysis was performed using Atlas.ti^®^ 22 [33].

The consolidated criteria for reporting qualitative research (COREQ guidelines) were followed [39].

### Research team and reflexivity

The female interviewer (DvN) is an experienced journalist and qualitative researcher. She had relatives with addiction problems. Although the first interview was the first time participants and interviewer met (after communicating through phone or e-mail), a relationship between the interviewer and participants developed, because of the intimacy of what was discussed and the long-term nature of the study. The second coder (research assistant) had no experience with addiction in her family. The research group as a whole consisted of a mix of people with and without experiential expertise on addiction in the family [33].

## Results

Five major themes were extracted from the data: (1) Informal support (effective, ineffective); (2) Educational support (effective, ineffective); (3) Healthcare support (effective, ineffective, COPMI); (4) Resilience factors; and (5) Developments over time.

For the relationship between the constructs of the SSICS-model, the interview questions, the coding, and the themes, see the Additional Table.

### Informal support

Most participants said they had experienced informal support, which came from several sources, depending on whom participants shared their experiences with: a parent without addiction problems, siblings, grandparents, their partner, friends, in-laws, or a friend’s parents. A few participants discussed the strain the addiction problems caused them – such as depressive symptoms – but did not disclose that the addiction problems of their relatives were the cause of their sadness or despair. A few participants did not tell anyone what they were going through.

#### Effective informal support

The most effective support was invariably the ability to discuss their experiences, provided some conditions were met: conversation partners were able to listen without judgment or unsolicited advice, they had participants’ best interest at heart, were able to sympathize with participants, or expressed recognition. This provided a sense of belonging.

A classmate and I were working for school and while we were working, I could just talk to him, about how things were going at home and tell him about it. He just said, “Yeah, okay, yeah, I get it.” It was very nice to have someone just listening at that moment.

(P7, 2nd interview, man, age range 23–27)

Most participants lived independently from their relatives with addiction problems, certainly by the end of the study, but reflecting on the time they had lived with their relatives, they felt practical support was beneficial. They could go to the houses of friends, neighbors, or grandparents, or live temporarily at their grandparents’ house. This allowed them to escape the home situation.

Our neighbor supported us immensely. We could always go to him. He helped us and we could always sleep and eat there. He was our constant… When he died, that was the worst thing of my life.

(P19, 1st interview, woman, age range 23–27)

The presence of pets was also described as effective. Dogs, cats, and horses provided security, warmth, and comfort.

My cat and dog always came and sat with me during an argument or when my mother was screaming. In turn, I took my dog out. That way the dog and I cared for each other and there was still love around.

(P9, 2nd interview, man, age range 23–27)

#### Ineffective informal support

Effective and ineffective support could occur side by side, depending on the person with whom participants were interacting. Friends, relatives, or others from the social environment giving judgment or unsolicited advice were perceived as ineffective.

The other person doesn’t have to offer a solution, just to listen. Sometimes I just want to say something but am immediately told: ‘You should do this, or do that’ or: ‘I would do this or that’. That doesn’t help me at all.

(P7, 2nd interview, man, age range 23–27)

Participants also did not appreciate too direct questions, explicit judgments, or jokes about relatives.

Someone from my in-laws asked out of the blue: ‘Does your father still drink that much?’ I don’t like that.

(P29, 3rd interview, male, age range 23–27)

Family members looking away – because they did not know how to act or wanted to avoid confrontation – were perceived as ineffective.

My father knew how bad the situation was. And yet, after the divorce, he wanted to take a neutral position and avoid confrontations with my mother while we were still living with her. In that respect, I didn’t experience much support.

(P26, 1st interview, woman, age range 23–27)

Some participants confided in someone from the family. If that trust was betrayed, the result was that they would not ask for help again. Other participants did not know how or who to turn to for help.

What could I have done? Call my parents [who both had addiction problems]? I mean: where do you go when you can’t handle it anymore?

(P6, 1st interview, woman, age range 23–27)

### Educational support

Half of the participants occasionally talked about their experiences with teachers and personal tutors. Others discussed the consequences, such as anxiety or depression, without discussing their relatives’ addiction problems. This included both secondary and higher education. Some participants in higher education spoke with internship supervisors about their experiences.

#### Effective educational support

One-third of the participants had positive experiences with teachers or personal tutors in higher education. Others had both positive and negative experiences. Most important were teachers having a listening ear.

You do have to pick the right people because some teachers understand, others don’t. She [a teacher] was just very sweet and very supportive. And also someone who regularly asked: hey, how are you doing?

(P2, 3rd interview, woman, age range 23–27)

Sometimes a single statement from a teacher was enough to get a student into a different mindset.

One teacher said he had figured out for himself that he had to do his best because he liked something, not because he thought he wasn’t good enough. That was exactly it. I didn’t think I was good enough either. From then on, I could focus on what I like and am interested in.

(P9, 1st interview, man, age range 23–27)

There was regular practical help from teachers and personal tutors in higher education, for example in setting achievable goals, learning to break down problems into smaller units, applying structure, and counseling for study delay. But teachers’ help often went beyond that. One participant who suffered from severe panic attacks upon entering the university found a teacher who was willing to meet her at a coffee bar instead. One participant even said she owed her life to teachers.

I didn’t want to live anymore. I was done with it. Then a teacher and two personal tutors sat with me for hours to see what they could do. They sought the right care and didn’t let go of me from then on. I’m still grateful for that.

(P26, 1st interview, woman, age range 23–27)

Participants who studied Social Work found the study program therapeutic. Supervision, self-reflection, writing, and discussing life stories ensured that disclosing a difficult family history had a place in education. Internship supervisors could also make a difference.

I was doing an internship in addiction treatment. I had told my team right away what was going on because I thought: these people know what it is like. I was often taken aside and asked: ‘Are you okay?’ I could just be open about it.

(P12, 3rd interview, woman, age range 23–27)

Looking back at secondary school, participants said they were helped with practical support from teachers, such as a quiet place at school to do homework. One teacher made it possible for a participant to continue attending the same school after she was placed out of home and moved to another city. Coincidentally, this teacher lived near her new place.

She said, ‘If you wait for me at that corner every morning, you can drive to school with me.’ So, I did and that way I could stay in school, with my friends. Thanks to her I finished high school.

(P10, 3rd interview, woman, age range 23–27)

#### Ineffective educational support

A few participants had only negative experiences with teachers or personal tutors. Some had both positive and negative experiences. Looking back to high school, participants who were truant, arguing, or underachieving experienced little interest from teachers in the causes of that behavior. Also, attention was lacking when their grades were good. In higher education, most students have annually changing personal tutors. This was experienced as ineffective because it prevented building a lasting relationship of trust. Teachers who had no genuine interest in the cause of study delay or even dropout were also perceived as non-supportive.

When I had sent an email that I was going to drop out, I got the question: ‘Are you sure?’ But that was a formality, I didn’t experience that as genuine interest.

(P17, 3rd interview, man, age range 28–33)

Some teachers did not understand the impact a relative’s addiction can have.

I told my study coach what was going on and she listened and occasionally asked me how things were going. But in my class, there was also someone with a mother with cancer. Teachers asked him every day how things were going at home and if he needed extra time to study for a test. Well, they didn’t ask me that. It seems like cancer is more important. But what I went through might have been just as difficult.

(P25, 1st interview, man, age range 18–22)

Some teachers gave negative feedback on the person which triggered unpleasant memories.

In the first year, a teacher said: ‘I don’t think you can make higher education.’ Well, that hit me hard. And then I had to prove that I could do it, over and over again. That’s something I inherited from that situation with my mother.

(P10, 1st interview, woman, age range 23–27)

Finally, one-third of the participants had no positive or negative experiences with teachers or personal tutors because they did not discuss their experiences at school or university. This could stem from shame. Also, some participants wanted school or university to be a space where their family problems were absent. This created a place where they felt ‘normal’.

### Healthcare support

At the start of our study, half of the participants received professional healthcare support. Help was sought from a wide variety of professionals (Table 2).

**Table 2:** Caregivers involved in participants’ lives (long-term and short-term)

Professionals	Number of respondents (throughout life to the end of our study)
Psychologists (CBT, EMDR, dialectical behavioral therapy, reattachment therapy, systemic therapy, couples therapy, grief counseling)	**18**
Police	**13**
General practitioner	**10**
Addiction care	**8**
Practice nurse	**6**
Youth care	**6**
Ambulance staff	**4**
12-step program	**3**
Funeral director	**3**
Social Work	**3**
Lifestyle training	**2**
Company doctor	**2**
Hospital psychiatric ward nurses	**1**
Peer group (sexual abuse)	**1**
Hospice volunteers	**1**
Physiotherapist	**1**
Chakra therapist	**1**
Haptonomist	**1**
Acupuncturist	**1**
Singing bowl therapist	**1**
Meditation	**1**
Mediator	**1**
COPMI-group	**1**
Halt Bureau^%^	**1**

^%^ Halt has a statutory duty to tackle criminal behavior among young people aged 12 to 23, without giving young people a criminal record

#### Effective healthcare support

Most participants were receiving Cognitive Behavioral Therapy (CBT) or Eye Movement Desensitization and Reprocessing (EMDR). An important characteristic of effective professional support was a long-term relationship of trust with a psychologist or a therapist, yet, at the same time, only a few participants had such long-term professional relationships. Within the therapies, several elements were described as effective. The most important element was learning to distinguish between accurate and inaccurate thoughts.

I always heard [from mother] that I was a bitch. So, I believed that. In therapy, we figured out a lot of problematic situations, and my therapist asked, “Who did this? Who did that?” It turned out it wasn’t me. Then I slowly began to believe that maybe I am not a bitch.

(P30, 3rd interview, woman, age range 23–27)

Other things that were also described as effective were learning to take other positions to examine a problem, developing empathy for the relative, and learning to put less pressure on themselves. A few times in the therapy sessions, participants were taught to recognize tension in their body, and then how to release it. Others emphasized how liberating it was to hear that they could choose for themselves, that they had not caused someone else’s addiction problems, and that they did not deserve the consequences.

Accept the pain but don’t accept that you deserve it. That one hit me like a sledgehammer.

(P16, 3rd interview, woman, age range 23–27)

Some participants specifically identified EMDR as effective, though they could not articulate why.

Before therapy, I felt so incredibly powerless. EMDR, that did help me a lot. I feel a lot more…peace.

(P2, 1st interview, woman, age range 23–27)

At the beginning of our study, the relatives of two participants were in treatment in addiction services. In our last interview session, that number had doubled to four participants. These were not the same participants. In all cases, there was a relapse of the relative during the research period. In one case, the brother of one of our participants developed a fairly stable recovery after relapse. The parents of this participant participated in a psychoeducational family program in which they learned that recovery usually does not happen in a straight line. They shared this knowledge with the participant which helped him handle it when that relapse came.

A few participants described meditation, reiki, or chakra therapy as effective because, with these therapies, they managed to keep negative energy from others away.

Several participants were taking medication: antidepressants, sleep medication, oxazepam, and/or medication for Attention Deficit Hyperactivity Disorder (ADHD). They experienced the medication as effective but were afraid of habituation. For most participants, taking medication was temporary.

#### Ineffective healthcare support

Despite these positive experiences, the participants reported more ineffective than effective professional support, for instance, because of having to work with different therapists, missing a connection with a therapist, long waiting lists – especially for trauma therapy –, and advice that they could not implement.

She said: ‘You have to let go of that situation with your brother, you are too worried, you make yourself too responsible for it. That is not your job.’ But how do you do that?

(P25, 4th interview, man, age range 23–27)

Some participants received many diagnoses and that stood in the way of good help.

My first diagnosis was mood disorder, then unspecified mood disorder, then depressive disorder, anxiety disorder, and only now, at diagnosis 5, PTSD. From the beginning, I said, I’ve been through a lot. I just wasn’t getting good help because it wasn’t the help I needed, which was EMDR.

(P1, 3rd interview, woman, age range 18–22)

Although EMDR worked for some, it did not work for everyone. Some could not bring themselves to mentally return to traumatic events, encountered memory problems, or mixed up traumatic situations. These participants also said EMDR did not teach them anything about coping strategies and did not help them move forward.

A few participants also came into contact with professionals who were perceived as lacking in sympathetic understanding.

I harmed myself severely and ended up in the hospital for that several times. A nurse once said to me: ‘We’ll just stitch it up without an anesthetic because you like pain, don’t you?’

(P16, 3rd interview, woman, age range 23–27)

In general, participants’ experiences with addiction care were not very positive. Addiction services were perceived as not treating the underlying problems of their relative such as trauma, not intervening when there was a crisis, not responding (adequately) to the emotional state of the participant, or even making the family feel responsible for the relative’s well-being.

The clinic wanted to use me as a tool to help my mother get rid of the addiction. So I was a tool, that was not to help me.

(P22, 1st interview, age range 23–27)

No participants joined a family recovery program. It was not offered, the relative was in treatment for only a short time, or the relative blocked family therapy.

In the family meeting, there was interest in us. Then we were heard, which I really liked. The therapist said that we could go into group therapy in the clinic because she noticed that I was still struggling with everything. But my mother didn’t want that. And, yeah, that was it.

(P30, woman, age range 23–27)

Finally, some participants chose a high deductible of their health insurance to keep monthly costs low. These costs could cause the participant to refrain from seeking help.

#### COPMI groups

At the first interview, 29 of the 30 participants did not know about the COPMI groups. Participants were informed about this preventive intervention by the interviewer, verbally and by email. During the follow-up interviews, no participants became interested in the COPMI groups. The reasons were that participants rated their situation as not serious enough, were afraid they had to deal with the problems of peers, found it too confronting to be in contact with others going through the same thing, or were convinced that they could not be helped at all. They were not able to disclose or were afraid of being overwhelmed by their own emotions.

Oh no, I don’t want that. Then I will be confronted with it even more. It seems so intense when everyone has the same experiences.

(P1, 1st interview, woman, age range 18–22)

To some, it felt unjust that they were the ones who had to seek help, while their relatives did not. Some participants were ashamed. They felt that their relative’s behavior also reflected on them. Others were already in treatment or had people in their lives who they could talk to. Some participants were afraid to run into acquaintances. A few said they were not heavily affected by their relatives’ addiction problems and for this reason, did not need a group intervention or peer-to-peer contact. One participant said she processed her past, another that she had no time. Looking back on their youth, two participants said that – even if they had known about and been interested in COPMI groups – they would not have been given parental permission when they were underage, and their parents also would not have paid their travel expenses.

### Resilience factors

Participants also found strength within themselves. Most participants were very goal-oriented. At the end of each interview, we asked about their goals for the next year. These goals could be small (painting the bathroom) or large (graduation from university). At the next interview, we found that these goals were often met. Some participants had a positive outlook on life or were able to laugh about situations they went through with their relatives. Some were motivated to do better than their relatives and actively planned a better life for themselves by investing in their education. A few were able to help themselves by getting tattoos. The meaning of the tattoo helped them just by looking at it.

That tattoo of a tree means: You are strong enough, you have the foundation, you have the roots to cope with everything.

(P13, 2nd interview, woman, age range 23–27)

Others engaged in sports or had a cultural outlet such as stand-up comedy. This made them feel physically well and boosted their self-confidence and through these activities, they sometimes found a community to which they belonged.

One participant felt empowered by a character in a video game.

A character in the videogame Halo, a super soldier, was the person I wanted to be. I clung to him. I saw him every day because I played Halo every day. He helped me through the day.

(P14, 2nd interview, man, age range 23–27)

Another participant found the many daily television episodes of Dr. Phil (an American talk show in which the host, Dr. Phil McGraw, gave his guests advice on their problems or lifestyle – often about addiction) effective because it became clear that addiction is common and the experiences of family members looked very much alike.

Watching that show every day I realized the problems at home have nothing to do with me, that was purely the addiction.

(P18, 1st interview, woman, age range 23–27)

A minority of the participants were not so strongly affected by living with their relatives with addiction problems. They said they did not have such a strong bond with the relative or did not associate his/her behaviors, insults, and humiliations with themselves.

### Developments over time

The support experiences of participants in our study show an erratic pattern. The themes identified in this paper – (in)effective informal, educational, and professional support – could co-occur throughout the study. For example, an understanding partner or teacher and struggling to find appropriate therapy could appear simultaneously.

Discussing their experiences with family, friends, and teachers was sometimes difficult but became easier as the participants grew older. Discussing these experiences was also a matter of practice.

You must practice talking about it. It’s a threshold you have to cross again and again. Even though I talk about it pretty easily now, there’s still tension attached to it …it’s not just any conversation topic, so to speak.

(P29, 3rd interview, man, age range 23–27)

Informal and educational support were described more frequently as effective than healthcare support. Nevertheless, the number of participants who sought healthcare support increased during this study. The search for help was often a long and winding road through various therapies and therapists. Men were less likely to seek and receive help than women. A long-term relationship of trust with a psychologist or a therapist was described as helpful, yet, at the same time, only a few participants had such long-term professional relationships. In contrast, contact with therapists was often short-term. Waiting lists meant that some participants had to wait a long time for suitable help. A minority of participants had contact with addiction care. Throughout this study, their number doubled from two to four, with six participants in total. Although three wrote an impact letter, no participant participated in a family recovery program. Experiences with addiction services were generally not rated positively.

The only targeted intervention for this group, the COPMI groups, was initially unknown. During the study (after being informed about the nature of the intervention) they did not become interested and did not visit the COPMI groups.

Participants experienced our study as beneficial which kept the retention rate high. They felt heard and seen. While their lives largely revolved around their relatives, they felt now there was time and interest for their story. Participation in the interview study resulted in some participants (including those who had not previously told others) being able to open up the conversation in the family; others were better able to talk about it with friends or classmates

Since we [participant and interviewer] have been in contact, I am much more open about the past and that connects me with others. If I tell what I’ve been through, then the other person also tells something about themselves. I found that many people have had a bit of a shitty life, and then you can talk to each other about that.

(P15, 2nd interview, woman, age range 23–27)

Some participants said they felt recognized because the impact of life with relatives with addiction problems was an important enough theme to study over several years. A few participants stated that contact with the interviewer, who was an expert by experience herself, gave them hope for a life in which they were no longer suffering from their experiences. Several participants had not realized until they started participating in this study how many others were in similar situations. This led to no longer feeling alone.

I always felt different from others, but knowing now that there are many more people like me, in the same situation, makes it more bearable. I feel normal now within a certain group, you know?

(P10, woman, age range 23–27)

A few participants asked if it was possible to continue the interviews and keep in touch. In several cases, this did happen.

#### Support needs

Over time, participants’ need for support shifted. In childhood, they needed nurturing and practical support. As they grew older, their needs moved to emotional support, comfort, understanding, and recognition. Most participants would have liked advice on how to deal with their relatives. Be involved or not? Give support or not? Flushing away alcohol or not? Professionals who were experts by experience could have made a difference, according to them. They also would have wanted advice made concrete, because they did not know how to ‘guard their boundaries’ or how to ‘take care of yourself’. A few said support should have been forced upon them or teachers should have forced them to talk.

If you know someone is in a bad place, don’t say, ‘If you want to talk…’, but ‘After this class we’ll talk.’

(P25, 2nd interview, man, age range 18–22)

Participants especially needed recognition of their distress by the relative. During our study, however, this recognition was only given twice.

She will always deny that she hurt us. She knows it, I am 100% sure of that, but she will never admit it.

(P19, first interview, age range 23–27)

Ultimately, participants wanted to be seen and heard, instead of all the attention being focused on their relatives.

Healthcare practitioners hurt me by being there for my sister [with addiction problems] and for my parents, but not for me. Everything was supposedly going well with me, even though I had scratches on my arms. Those practitioners made me feel rejected again. As if I did not belong in the family. I wanted to be seen and heard too! See me. That’s all. At the end of your thesis will be a sentence that sums everything up: Please, see me.

(P16, 3rd interview, woman, age range 23–27)

## Discussion

Our study aimed to examine (1) AFMs’ experiences with informal and professional support in coping with the impact of relatives’ addiction problems and (2) how these experiences evolved over time.

First, we found that informal support was more often experienced as effective than professional support. This finding contradicts Orford et al. [2] who state that informal support for AFMs in general is “often perceived as unsupportive, critical or even overtly hostile to the AFM’s position” (p74). It is, however, in line with studies that show that high levels of social support after a variety of traumas (for example child abuse) have been associated with better health outcomes among student populations [40, 41]. Furthermore, a few of our participants benefited from sports, meditation, reiki, or chakra therapy, and also from cultural activities, such as stand-up comedy. These forms of support were not used in the therapeutic interventions that the students had received. The scientific literature on physical activity and body-oriented therapy is scarce and diverse, coming from a wide range of disciplines and therapeutic orientations, including sport and exercise therapy, yoga, qigong, and t’ai chi, dance movement therapy, psychomotor therapy, somatic experiencing, acupoint therapies, or touch therapies [42, 43]. Although body-oriented psychotherapy interventions are effective in different populations and settings [44, 45], treatments are neither standardized nor systematically evaluated [44, 46]. The same is true for creative and art therapies [47]. Further research should establish the acceptability and effectiveness of these forms of support for AFMs, discover working mechanisms, and identify how these interventions could be offered within and outside of a therapeutic setting.

Regarding professional help, we differentiated between educational and healthcare support. Participants valued educational support better than healthcare support, although half of the participants did not discuss their experiences at school or university. A general reluctance amongst higher education students to disclose their mental health problems – stemming from fear of stigma, or lack of knowledge about mental health (on their part and also on the part of teachers) – has been established before [3, 48].

Research on healthcare support for AFMs focuses primarily on prevention programs and specific interventions to improve the health and well-being of family members affected by relatives’ addiction problems. These interventions, however, are limited in number and scope [15, 49, 50]. At the same time, research has paid little attention to support for AFMs from regular mental health services, even though this is precisely what the participants in our study increasingly looked for and received. We found that healthcare treatment tended to diagnose participants with Post Traumatic Stress Disorder (PTSD), depression, and anxiety. Participants were mainly treated with CBT and EMDR, both considered first-choice treatments for PTSD by the World Health Organization [51]. Little research has been done on the effectiveness of these PTSD treatments related to child abuse or trauma related to life with relatives with addiction problems because exclusion criteria for research studies frequently included suicide attempts or ideation, self-harm, or problematic substance use [52, 53]. These problems are particularly prevalent in our group [8]. Future research should generate more knowledge about the efficacy of trauma therapy for AFMs.

Our participants did not join family recovery programs in addiction care, although some relatives were in contact with addiction treatment professionals. Much research has been done on the importance of family engagement in relatives’ recovery [54]. However, the perspective of those family members remains understudied [55]. Even though only some of our participants were in contact with addiction services, our study contributes to the research on the involvement of family members in addiction recovery.

Participants were not interested in the COPMI groups. A group intervention for AFM peers seemed to scare the participants. This fear is of interest, especially since it has been established that peer support in mental healthcare is effective, especially for persons with the same lived experiences [56, 57]. To our knowledge, no research has been conducted before on the barriers for young adult AFMs to attend peer support groups.

We found several individual, family, and community factors related to resilience. Some participants had an optimistic nature or were determined to create a better future for themselves, which contributed to their resilience. Although not mentioned by participants, it is safe to say that students in higher education are intelligent. Their understanding of problems and ability to solve some problems contributed to their resilience. These factors have been described before [12, 58, 59]. Less known are pets and tattoos as resilience-enhancing factors. Previous research showed that people with lower levels of trust in others, greater fear of rejection, and higher psychological strain tend to have strong emotional attachment to pets [60]. Obtaining tattoos has been found to correlate with the acceptance of self and others and to support a healing process [61]. We also found that family factors, such as having a trusting relationship with another family member or significant other, and community factors, such as support from teachers and neighbors contributed to resilience. This has also been described before [12, 58].

The concept of resilience as used in the literature on AFMs is related to that of (family) recovery as used in the addiction literature. There are four components of (family) recovery capital. Social capital (personal relationships), physical capital (possessions and money), human capital (skills, positive health, the will to achieve something, educational attainment, intelligence), and cultural capital (values and beliefs) [62]. As with resilience, (family) recovery is increasingly seen as a non-linear process rather than a state, with many pathways [55, 63]. Our study contributes to the literature on (family) recovery by showing that family members go through their own recovery process and follow their own pathways, even if their relatives do not.

Second, concerning the longitudinal nature of our study, we found that the need for professional support among participants increased, that participants did not become interested in the targeted intervention of the COPMI groups and that support needs shifted during their life course. LQR proved to be a suitable method to determine this. LQR may be applied to understand any human experience and is particularly well suited for studying transition periods and behavioral changes across time. LQR may also be applied to inform the development of interventions and may be used to understand if a policy or program was effective [64].

### Strengths and limitations

This is the first longitudinal qualitative study that examined AFMs’ access to support, the quality of received support, and the kind of support that was desired but not received. By using this longitudinal qualitative method, development and change have been made visible, and participants opened up more towards the interviewer. Another strength is the study sample, which was diverse in terms of gender, study program, study year, relatives’ type of substance use, relationship to the relative, and the degree of disclosure. Participation rates were also high; 93% participated in at least two interviews. However, a possible limitation concerns selection bias. Participants selected themselves by providing contact details for further research after participating in a quantitative survey, possibly resulting in the inclusion of those who strongly identified as AFM. Similarly, participants were predominantly of Dutch ethnic origin, while the RUAS student population is more diverse. Attempts to include participants from other ethnic backgrounds proved unsuccessful.

### Recommendations

The importance of lending a listening ear or offering a helping hand for people suffering from their relatives’ addiction problems is shown in our study. This can be done by anyone: family, friends, colleagues or neighbors.

To prevent study delay or study dropout, trauma-informed approaches in schools and universities could be implemented. However, trauma-sensitive education is still in its infancy, initiatives are poorly documented and evaluated, and not evidence-based [3, 65, 66]. Therefore, we recommend that teachers and personal tutors, through co-creation with AFMs, build knowledge, train teams, and staff to identify students suffering from the addiction problems of their family members, discuss these experiences, gain knowledge of services, and refer them adequately. Moreover, universities of applied sciences train for all kinds of professions that interact with AFMs in practice, such as nurses, midwives, ambulance workers, (ortho)educators, social workers, teachers, lecturers, and human resource workers. There is no or knowledge transfer on AFMs in these education programs. Knowledge and competence development on the nature and prevalence, identification, recognition, and support of AFMs should be included in the curricula of relevant education programs.

Healthcare professionals should be better informed about the experiences and needs of AFMs. This would prevent negative reactions and promote empathy from professionals. Therefore, it is recommended to train professionals specifically for the treatment of young people with relatives with addiction problems. Addiction treatment professionals should establish, in discussion with AFMs, whether it is also in the interest of these AFMs to be involved in the treatment of their relatives and under what conditions. If AFMs want to be involved, they should also be supported in their own right.

Additionally, we advise providers of COPMI-group preventive interventions to take note of the concerns of young adult COPMI as expressed in our study and take these concerns into account in their communications. We also advise offering individual trajectories, to prevent AFMs from avoiding these interventions for fear of a group intervention. Further research into the reach, effectiveness, costs, and benefits of COPMI-interventions is needed as well as the reluctance of professionals to refer children and adolescents to these interventions.

Professionals who are experts by experience could make a difference, according to our participants. Healthcare practitioners who are AFMs could make themselves known to clients or patients with similar experiences, something which would reduce stigma and shame.

We also recommend broadening the professional support offered to AFMs from regular therapies, such as EMDR and CBT, to include body-oriented and cultural interventions. What may be effective is a toolbox of effective interventions from which anyone can extract something that might help them at different times in their life.

Finally, we recommend that those who (have) struggle(d) with addiction discuss the impact of their addiction problems with their family members. Acknowledgment of their pain can give AFMs the recognition they need.

## Conclusions

Most young adult AFMs in our study received support from a variety of people in their social environment and from a wide range of professionals. They described informal and educational support as more effective than professional support. Participants were mainly treated with CBT and EMDR. Body-oriented therapy was remarkably absent. The number of participants seeking healthcare support increased during our research. For most, this was a journey of trial and error. Support that was needed shifted during their lives from practical to emotional support. Participants were very much in need of recognition by their relatives for harm done. Unfortunately, this rarely happened.

## Electronic supplementary material

Below is the link to the electronic supplementary material.

Supplementary Material 1

Supplementary Material 2

## Data Availability

The data supporting this study’s findings (interview transcripts) are not publicly available because they contain information that could compromise the privacy of research participants but are available from the corresponding author upon reasonable request.

## Abbreviations

ACOA: Adult Children of Alcoholics
AFM: Affected Family Member
CBT: Cognitive Behavioral Therapy
COPMI: Children Of Parents with Mental Illness and/or addiction
CRAFT: Community Reinforcement and Family Training
EMDR: Eye Movement Desensitization and Reprocessing
HUAS: the Hague University of Applied Sciences
LQR: Longitudinal Qualitative Research
PTSD: Post Traumatic Stress Disorder (PTSD)
RUAS: Rotterdam University of Applied Sciences
SSICS: Stress-Strain-Information-Coping-Support-model
