# Supplementary material for: ‘Please, see me’; Informal and professional support of students with relatives with addiction problems: a three-year longitudinal qualitative study
Source: BMC Public Health. 2024 Nov 8;24:3092. doi: 10.1186/s12889-024-20531-8 (PMC11545315; doi:10.1186/s12889-024-20531-8)
Supplement: Supplementary file 1 — Supplementary Material 1 [file 12889_2024_20531_MOESM1_ESM.docx]

**Appendix List of topics for the interviews**

| **Introduction**  Introducing myself, explaining purpose of research, naming experiential expertise, asking how participant feels, offering a drink. If necessary coffee/tea-break to reduce stress. |
| --- |
| **About the student**  What study, year of study, study delay, age, ethnic background, relationship to relative, type of addiction, other family members with addiction?, living situation  Why do you want to participate in this study? |
| **About the family**  You have indicated that you have one or more family members with addiction. Because of which relative are you participating in this study?  How many people in your family with addiction?  Family composition (parents together/separated, brothers/sisters) |
| **About the relatives’ addiction**  What addictions are you confronted with in your nuclear family?  When did you begin to realize that something was going on with your family member's/relatives' use/behavior?  What do you think is the reason for the addiction?  Does your relative with addiction work? Does he/she attend school? Otherwise active? |
| **Stress**  Can you tell me something about life with your relative(s)/relatives’ addiction?  What does your relative's addiction behavior look like?  Can you outline a few events you link to the addiction?  What is the worst thing about your relative’s addiction?  Have you experienced violence due to the addiction? Have you ever been in mortal danger? What about others in your family?  Do you experience other effects of your family member's addiction? (e.g. financial problems/debts)  How does/did everyday life in your household look like? (housework, admin, caring for younger brothers/sisters/)  Do you see any positive aspects of living with someone else's addiction? |
| **Strain**  Can you describe the effects your relative’s addiction has on you? (health issues that you relate to your family member's addictive behavior? (physical, mental))?  Are you sleeping well? (if not, what sleeping problems do you have? Nightmares?)  Do you use alcohol/drugs yourself? To what extent? Do you gamble/game? To what extent?  How do you feel about your relative(s)? |
| **Social life**  What is/was your family life like? Did you do things together? Eating together, for example? Holidays?  How is your relationship with the other family members?  Are you in a relationship? m/f?  Do you have a side job? Where? How many hours?  What do you do when you are free from school/work? (Do you have friends? Do you go out? Often? Do you do any sports? Other leisure activities? (e.g. creative)) |
| **About the study**  What study course do you follow? How do you like it?  What does your study mean to you?  Do you have study delay?  Is this your first study?  How are you doing in school? Does the situation in your family influence your studies? How?  Has addiction in your family ever come up at school? With whom? How did it go?  Have you ever been bullied? For example in high school? |
| **Coping**  People cope very differently with a relative with addiction. How do you deal with it? (Tolerating, withdrawing, controlling, angry, taking over responsibilities?)  When do you find it relatively easy to deal with? When do you find it more difficult to cope? |
| **Disclosure**  Do you ever talk to others about the addiction of your relative(s)?  To whom?  How do you feel when you talk about it? |
| **Support**  What do you do when you feel desperate/alone/in doubt?  Have you ever looked for help? Where?  Have you ever received help or support regarding the situation in your family?   - From family/friends/partner? - From neighbors? - From school/internship? - Professional support? - Peers?   If yes, was that support helpful?  If not, what kind of support did you need?  Did other family members receive help or support? If so, from whom?  Have you ever heard of COPMI-groups (i.e. a support group for children 0-23 yo, with parents with mental health or addiction problems)? If so, how? Through school/doctor? Otherwise? If not (after explanation): could this be something for you? |
| **Future**  What expectations do you have about the future?  What do you want to have accomplished before we see each other again next year? |
| **Closing**  Are there any topics we didn't discuss that are important to you when it comes to life with someone else's addiction?  What did you think of this interview? How do you feel about it?  How do you feel now? (relieved, tired, sad) |
