# Supplementary material for: ‘Please, see me’; Informal and professional support of students with relatives with addiction problems: a three-year longitudinal qualitative study
Source: BMC Public Health. 2024 Nov 8;24:3092. doi: 10.1186/s12889-024-20531-8 (PMC11545315; doi:10.1186/s12889-024-20531-8)
Supplement: Supplementary file 2 — Supplementary Material 2 [file 12889_2024_20531_MOESM2_ESM.docx]

**Additional Table From construct to themes**

| Construct Support in SSICS model | Interview questions | Codes deductively from the model | Codes inductively from the data | Themes |
| --- | --- | --- | --- | --- |
| (1) Informal support (kin, non-kin)  (2) Professional support | What do you do when you feel desperate/alone/in doubt?  Have you ever looked for help? Where?  Have you ever received help or support regarding the situation in your family?   - From family/friends/partner? - From neighbors? - From school/internship? - Professional support? - Peers?   If yes, was that support helpful?  If not, what kind of support did you need?  Did other family members receive help or support? If so, from whom?  Have you ever heard of COPMI-groups (i.e. a support group for children 0-23 yo, with parents with mental health or addiction problems)? If so, how? Through school/doctor? Otherwise? If not (after explanation): could this be something for you? | Informal support   - family - peers - partner - social (neighbours, parents friends) - work - other   Professional support   - Healthcare professional - Other | Educational support   - secondary school - university - internships   Support other   - selfhelp - online, television - animals | Informal support (helpful, non-helpful);  Support in education (helpful, non-helpful);  Healthcare support (helpful, non-helpful, COPMI groups);  Self-help strategies;  Support needs |
